# Impact of arm movement strategies on emotional state and gait outcomes during height-induced postural threat in healthy children compared to young adults

**DOI:** 10.1007/s00221-025-07112-w

**Published:** 2025-06-03

**Authors:** Anna M. Wissmann, Mathew W. Hill, Thomas Muehlbauer, Johanna Lambrich

**Affiliations:** 1 Division of Movement and Training Sciences/Biomechanics of Sport, University of Duisburg-Essen, Gladbecker Str. 182, 45141 Essen, Germany; 2 Center for Physical Activity, Sport and Exercise Sciences, Coventry University, Coventry, UK

**Keywords:** Postural control, Gait, Locomotion, Anxiety, Perception, Upper body strategy, Age

## Abstract

Empirical evidence indicates that height-induced postural threat as well as the restriction of arm movements lead to detrimental effects on walking performance. However, it is unclear whether the deteriorations are more pronounced in children (i.e., due to incomplete maturation) compared to young adults. This study investigated the effects of different arm movement strategies on subjective and objective indicators related to walking at or above ground-level in children compared to young adults. Twenty-nine children (age: 11.1 ± 0.3 years) and 26 young adults (age: 24.0 ± 4.7 years) walked five meters at self-selected speed on ground-level (no threat) and 80 cm above ground-level (threat) with free and restricted arm movements. Walking outcomes (i.e., gait speed, cadence) were measured and used as objective markers. Self-reported emotional state outcomes (i.e., balance confidence, fear of falling, perceived safety, conscious balance processing) were assessed and used as subjective indicators related to walking. Children significantly differed from young adults in objective and subjective outcomes related to gait by showing no decrease in walking cadence from the no threat to the threat condition (irrespective of arm movement condition) and a decrease in perceived safety when walking with restricted compared to free arm movements (irrespective of threat condition). The findings extend previous research related to postural threat and arm restriction while walking in young adults and provide new insights into understanding how children behave under these conditions.

## Introduction

Numerous studies (Carpenter et al. 2001; Laufer et al. 2006; Huffman et al. 2009; Hill et al. 2023, 2024) have examined the influence of height-induced postural threat during upright stance. Studies demonstrated that standing at an elevated height compared to ground-level consistently causes a postural ‘stiffening reaction’ (Adkin and Carpenter 2018). This is expressed by a decrease in centre of pressure (CoP) amplitude metrics and a concurrent increase in CoP frequency parameters (Carpenter et al. 2001; Huffman et al. 2009; Adkin and Carpenter 2018). Additionally, the influence of height-induced postural threat during standing on emotional state outcomes such as fear and anxiety is also well studied (Adkin and Carpenter 2018). Specifically, self-reported emotional responses indicate an increase in fear of falling, perceived instability (i.e., decreased perceived safety) and conscious balance processing when people transfer their stance position from ground-level to above the ground (Hill et al. 2023, 2024). While the previously described changes in static balance tasks have already been well investigated, there are comparatively few studies analysing such threat-related adaptations in dynamic balance tasks (Brown et al. 2002; Gage et al. 2003; McKenzie and Brown 2004; Ellmers and Young 2018; Kluft et al. 2020; Lambrich et al. 2025). Together, these studies demonstrated threat-related deteriorations such as a decrease in walking speed parameters. The existing literature on changes in dynamic postural control has predominantly focussed on young (Brown et al. 2002; Gage et al. 2003; McKenzie and Brown 2004; Ellmers and Young 2018; Lambrich et al. 2025) and older (Brown et al. 2002; Gage et al. 2003; McKenzie and Brown 2004; Kluft et al. 2020) adults, while comparable adaptations in children remain unexplored.

Children aged 6 to 12 years may be more susceptible to the effects of threatening stimuli on postural control due to maturational deficits in postural control mechanisms (Shumway-Cook and Woollacott 1985; Hirabayashi and Iwasaki 1995; Orendorz-Fraczkowska and Kubacka 2019; Sinno et al. 2021) and ongoing development of emotional processing capacities (De Sonneville et al. 2002; Perlman and Pelphrey 2011). Consequently, children may lack the motor and cognitive skills to effectively suppress threat-related stimuli during dynamic movement tasks performed at height. Evidence from cross-sectional studies (Stins et al. 2009; Hill et al. 2024) suggests that children exhibit a behavioural pattern more similar to highly anxious individuals when *standing* at height, such as an increase in CoP amplitude, rather than the decreased amplitude typically observed (Adkin and Carpenter 2018). However, these findings do not allow direct conclusions to be drawn about how dynamic movement tasks at height affect children’s postural control. Thus, there is a need for research to investigate how dynamic movement tasks at height– and the emotional responses they trigger– influence dynamic postural control in children.

Further studies (Hebert-Losier 2017; Boström et al. 2018; Hill et al. 2019; Objero et al. 2019) showed that the restriction of arm movements can negatively affect static and dynamic postural control in addition to standing and walking at height. In particular, an increase in CoP amplitudes in static balance tasks (Hebert-Losier 2017; Hill et al. 2019; Objero et al. 2019) and a decrease in gait velocity in dynamic balance tasks (Boström et al. 2018; Hill et al. 2019) were observed under conditions of restricted compared to free arm movements. Presumably this is explained by the fact the restriction of arm movements reduces the distribution of body mass and thus reduces the moment of inertia, which affects the stability of the postural control system (Hill et al. 2019). If arm movements are restricted, the arms cannot be used to generate restoring torques and reduce the body's angular momentum (Roos et al. 2008), nor can they be used as a counterweight to shift the centre of mass in the opposite direction of instability (Marigold et al. 2003). In contrast, free arm movements have a compensatory effect, as they can attenuate the deterioration in postural control caused by increased balance task difficulty (Johnson et al. 2023b) or by altered sensory conditions (Johnson et al. 2023a). If these considerations are transferred to height-induced decrements in postural control, it seems plausible to assume that these are less pronounced in test conditions with free than with restricted arm movements. To date, only two studies (Hill et al. 2023; Lambrich et al. 2025) have investigated the influence of different arm movement strategies on behavioural and emotional responses to height-induced postural threat. First, Hill et al. (2023) demonstrated compensatory effects, showing that young adults exhibited significantly lower CoP amplitude, fear of falling, and perceived instability (higher perceived safety) at height when their arms were free compared to when they were restricted. Second, Lambrich et al. (2025) found that walking with free compared to restricted arm movements at height was associated with higher balance confidence and faster step time. Although both studies have provided valuable insight, they were conducted exclusively in young adults, without considering potential influences of developmental differences in children's postural control mechanism. Thus, we sought to extend the current level of knowledge by comparing these effects across different age groups (i.e., children vs. young adults).

Therefore, the current study compared the effects of free and restricted arm movements on subjective and objective indicators related to walking on ground (no threat) or at height (threat) between children and young adults. Irrespective of arm movement, we assumed from the work (Brown et al. 2002; Gage et al. 2003; Hill et al. 2019, 2023; Muehlbauer et al. 2022; Lambrich et al. 2025) conducted thus far that (i) postural threat would elicit detrimental effects on subjective (i.e., emotional state) and objective (i.e., gait data) outcomes related to walking, (ii) the effects would be more pronounced when arm movements were restricted, and (iii) the effects would be larger in children compared to young adults.

## Material and methods

### Participants and sample size estimation

Sample size estimation for repeated measures analysis of variance (ANOVA) were conducted from previous studies that examined the impact of height-induced postural threat (Brown et al. 2002) or arm movements (Roos et al. 2008; da Silva Costa et al. 2023) on dynamic postural control. This estimation was performed using G*Power software version 3.1.9.7 (Faul et al. 2007) and revealed that a minimum of 38 participants (*n* = 19 per age group) would be necessary to identify statistically significant interaction effects of arm restriction on gait outcomes when exposed to postural threat (input parameters: effect size [*f*] = 0.25, significance level [*α*] = 0.05, power [1 − *β*] = 0.80, number of groups = 2, number of measurements = 4, correlation among repeated measures *r* = 0.20). A total of 55 participants volunteered for this study (Table 1). Children were recruited from a secondary school in Essen, North-Rhine-Westfalia, Germany. Young adults were enrolled from the student population of the host institution. All participants were free of musculoskeletal dysfunction, neurological impairment, or orthopaedic disorder and had no serious concern about visual height intolerance. Prior to conducting the experiment, all participants (as well as the children’s parents) gave their written informed consent and the Human Ethics Committee at the University of Duisburg-Essen, Faculty of Educational Sciences approved the study protocol (approval number: EA-PSY9/24/25032024).

**Table 1:** Characteristics of the participants by age group

Characteristic	Children	Young adults
Sample size (*n*)	29	26
Gender (females; *n*)	18	15
Age (years)	11.1 ± 0.3	24.0 ± 4.7
Stature (cm)	152.1 ± 6.1	174.0 ± 3.0
Mass (kg)	40.0 ± 7.0	74.0 ± 15.1
Body mass index (kg/m^2^)	17.2 ± 2.3	23.8 ± 3.4
vHISS	1.1 ± 1.7	0.7 ± 1.3

*vHISS* Visual height intolerance severity scale, i.e., a 10-item questionnaire that provides a severity score ranging from 0 (‘Not at all’) to 13 (‘Severe’)

### Experimental procedures

**Fig. 1:**
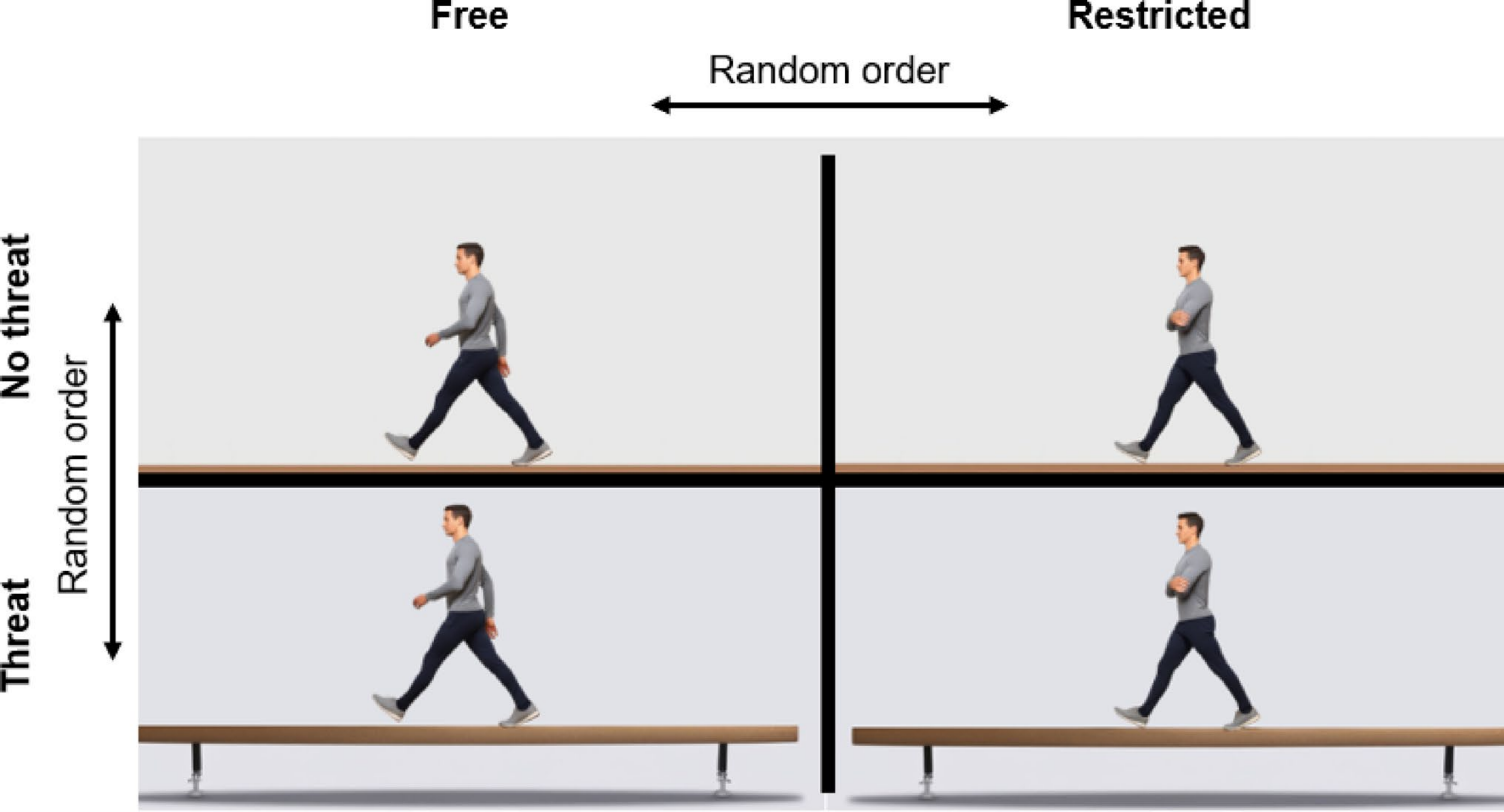
Schematic diagram of the postural threat (no threat vs. threat) and arm movement (free vs. restricted) conditions

Participants completed dynamic balance tasks by walking a distance of five meter under two different arm movement conditions: (i) hands clasped in front of the body at waist level ("restricted" arm movement), and (ii) arms freely moving ("free" arm movement). Each participant completed one trial per arm condition at ground level ("no threat") and 80 cm above ground level (“threat”). The order of the four conditions was randomised, both in terms of the arm condition and the walking height. For the “no threat” condition, the walkway (length: 500 cm, width: 10 cm) was marked out on the floor with adhesive tape. In the “threat” condition, a commercially available balance beam, as utilized for gymnastics, was used. This wooden beam had a length of 500 cm and a width of 10 cm and was mounted on supports 80 cm above the floor (Fig. 1). Access to the balance beam was ensured via a box staircase with a podium. Prior to data collection, participants completed a practice trial to familiarise themselves with the individual test conditions. For all trials, participants were instructed to walk forwards at a self-selected speed while wearing their own sport shoes and without a safety harness. Each walk was initiated and terminated at least one meter before and after the 5 m walkway to allow sufficient space for acceleration and deceleration. In other words, the distance from 0–1 m was used for acceleration, from 1 to 6 m for assessment, and from 6–7 m for deceleration. For walking above ground, this was ensured by a box staircase with a podium at both the start and end of the balance beam.

### Assessment of emotional state outcomes

Immediately before each trial, participants rated their confidence in maintaining balance while standing at the beginning of the walkway using a visual analogue scale (VAS) ranging from 0 (‘Not at all confident’) to 10 (‘Completely confident’) (Davis et al. 2009; Zaback et al. 2016). After each trial, participants rated their fear of falling during the trial on a VAS ranging from 0 (‘Not at all fearful’) to 10 (‘Completely fearful’) (Huffman et al. 2009; Hill et al. 2023). They also rated their perceived safety during the experiment on a VAS, where 0 stood for ‘Completely safe’ and 10 for ‘So unsafe that I would fall’ (Castro et al. 2019; Ellmers et al. 2021). In addition, participants completed a 4-item questionnaire to assess conscious processing of balance. The items included: (1) ‘I always try to think about my balance when I perform this task’. (2) ‘I am aware of how my mind and body are functioning when performing this task’. (3) ‘I am aware of how I look when performing this task’. (4) ‘I am concerned about my movement style when performing this task’. Each item was scored on a scale from 1 (‘strongly disagree’) to 6 (‘strongly agree’) (Ellmers and Young 2018). The total score of these four questions (between 4 and 24) was used for the analysis, with higher scores indicating greater conscious processing of balance.

### Assessment of gait outcomes

The walking time (s) was manually recorded with a stopwatch (resolution: 0.01 s) by the same graduated student to ensure accuracy of the start and stop for each trial, based on visual observation. The time needed for 5 m walking distance was used to calculate gait speed (m/s). The number of steps (*n*) was counted visually by the same experimenter while the participants walked the 5-m measuring distance (i.e., 1–6 m), leaving a one-metre space before (i.e., 0–1 m) and after (i.e., 6–7 m) the walkway to allow sufficient distance to accelerate and decelerate. Cadence (*n*/s) was calculated by dividing the step number (i.e., counted from heel contact of one foot to heel contact of the other foot) by the time needed for 5 m walking distance.

### Statistical analyses

The data were analysed using JASP version 0.17.3 (Amsterdam, The Netherlands). Before performing parametric analyses, the assumptions of normality (Shapiro–Wilk test) and homogeneity of variance/sphericity (Mauchly test) were tested and met. A series of separate mixed model ANOVAs were performed to examine the between-subject effect of age group (× 2 [children vs. young adults]) and the within-subject effects of postural threat (× 2 [no threat vs. threat]) and arm condition (× 2 [free vs. restricted]). When significant interactions or main effects were identified, post-hoc tests with Bonferroni-adjusted alpha levels were used to localise specific differences. Effect sizes for ANOVAs were expressed as partial eta-squared (${\eta }_{p}^{2}$) and categorised as small (0.02 ≤ ${\eta }_{p}^{2}$  ≤ 0.12), medium (0.13 ≤ ${\eta }_{p}^{2}$  ≤ 0.25), or large (${\eta }_{p}^{2}$≥ 0.26). Pairwise comparisons were indicated with Cohen's *d* (Cohen 1988) and interpreted as trivial (0 ≤ *d* ≤ 0.19), small (0.20 ≤ *d* ≤ 0.49), moderate (0.50 ≤ *d* ≤ 0.79), or large (*d* ≥ 0.80). The alpha level for all tests was set a priori at *p* < 0.05.

## Results

Table 2 shows the descriptive statistics consisting of group mean ± standard deviation values and Table 3 presents the ANOVA outputs (i.e., inferential statistics) for all assessed variables.

**Table 2:** Descriptive statistics showing group mean ± standard deviation values for all emotional state and walking outcomes for children and young adults by threat conditions (no threat vs. threat) and arm movement strategies (free vs. restricted)

Parameter	Arm strategy	Children (*n* = 29)		Young adults (*n* = 26)	
		No threat	Threat	No threat	Threat
*Emotional state outcomes*					
Balance confidence (0–10)	Free	9.1 ± 1.0	7.8 ± 1.7	9.7 ± 0.6	8.2 ± 1.8
	Restricted	8.6 ± 1.4	7.2 ± 1.7	9.6 ± 0.7	7.5 ± 1.6
Fear of falling (0–10)	Free	1.4 ± 1.6	3.0 ± 2.0	0.1 ± 0.3	1.2 ± 1.4
	Restricted	2.2 ± 1.8	3.6 ± 2.1	0.2 ± 0.5	1.7 ± 1.9
Perceived safety (0–10)	Free	1.7 ± 1.6	3.0 ± 1.9	0.3 ± 0.6	2.0 ± 1.7
	Restricted	2.1 ± 1.7	3.5 ± 2.1	0.6 ± 0.9	1.7 ± 1.3
Conscious processing (4–24)	Free	13.8 ± 3.5	15.5 ± 2.6	11.7 ± 4.5	13.1 ± 3.6
	Restricted	14.4 ± 3.7	15.8 ± 2.6	12.1 ± 4.2	13.3 ± 3.8
*Walking outcomes*					
Gait speed (m/s)	Free	1.37 ± 0.27	1.10 ± 0.28	1.31 ± 0.37	1.03 ± 0.37
	Restricted	1.33 ± 0.29	1.04 ± 0.22	1.21 ± 0.47	1.00 ± 0.33
Cadence (*n*/s)	Free	1.63 ± 0.19	1.68 ± 0.24	1.75 ± 0.27	1.62 ± 0.32
	Restricted	1.65 ± 0.22	1.59 ± 0.19	1.76 ± 0.47	1.60 ± 0.32

**Table 3:** Inferential statistics showing main and interaction effects of the repeated measures ANOVA for emotional state and walking outcomes

Parameter	Group (children vs. young adults)	Threat (no threat vs. threat)	Arm (free vs. restricted)	Threat × Arm	Group × Threat	Group × Arm	Group × Threat × Arm
*Emotional state outcomes*							
Balance confidence (0–10)	***F* = 5.461** ***p (η***_**p**_^**2**^**) = 0.023 (0.09)**	***F* = 56.409** ***p (η***_**p**_^**2**^**) < 0.001 (0.52)**	***F* = 13.953** ***p (η***_**p**_^**2**^**) < 0.001 (0.21)**	*F* = 3.456 *p (η*_p_^2^) = 0.069 (0.06)	*F* = 1.266 *p (η*_p_^2^) = 0.266 (0.02)	*F* = 0.473 *p (η*_p_^2^) = 0.494 (0.01)	*F* = 3.456 *p (η*_p_^2^) = 0.069 (0.06)
Fear of falling (0–10)	***F* = 24.944** ***p (η***_**p**_^**2**^**) < **0**.001 (**0**.32)**	***F* = 59.519** ***p (η***_**p**_^**2**^**) < **0**.001 (.53)**	***F* = 13.885** ***p (η***_**p**_^**2**^**) < **0**.001 (0.21)**	*F* = 0.378 *p (η*_p_^2^) = 0.541 (0.01)	*F* = 0.413 *p (η*_p_^2^) = 0.523 (0.01)	*F* = 2.294 *p (η*_p_^2^) = 0.136 (0.04)	*F* = 1.818 *p (η*_p_^2^) = 0.183 (0.03)
Perceived safety (0–10)	***F* = 16.303** ***p (η***_**p**_^**2**^**) < 0.001 (0.24)**	***F* = 65.448** ***p (η***_**p**_^**2**^**) < **0**.001 (0.55)**	*F* = 3.041 *p (η*_p_^2^) = 0.087 (0.05)	*F* = 1.175 *p (η*_p_^2^) = 0.283 (0.02)	*F* =.014 *p (η*_p_^2^) = 0.907 (0.00)	***F* = 4.289** ***p (η***_**p**_^**2**^**) = 0.043 (0.08)**	*F* = 1.967 *p (η*_p_^2^) = 0.167 (0.4)
Conscious balance processing (4–24)	***F* = 6.777** ***p (η***_**p**_^**2**^**) = 0.012 (0.11)**	***F* = 31.832** ***p (η***_**p**_^**2**^**) < 0.001 (.38)**	*F* = 3.887 *p (η*_p_^2^) = 0.054 (0.07)	*F* = 0.933 *p (η*_p_^2^) = 0.338 (0.02)	*F* = 0.194 *p (η*_p_^2^) = 0.661 (0.00)	*F* = 0.198 *p (η*_p_^2^) = 0.659 (0.00)	*F* = 0.014 *p (η*_p_^2^) = 0.906 (0.00)
*Walking outcomes*							
Gait speed (m/s)	*F* =.801 *p (η*_p_^2^) = 0.375 (.02)	***F* = 71.586** ***p (η***_**p**_^**2**^**) < 0.001 (.58)**	***F* = 11.988** ***p (η***_**p**_^**2**^**) = 0.001 (0.18)**	*F* = 0.187 *p (η*_p_^2^) = 0.667 (0.00)	*F* = 0.286 *p (η*_p_^2^) = 0.595 (0.01)	*F* = 0.200 *p (η*_p_^2^) = 0.656 (0.00)	*F* = 1.472 *p (η*_p_^2^) = 0.230 (0.03)
Cadence (*n*/s)	*F* = 0.499 *p (η*_p_^2^) = 0.483 (0.01)	***F* = 6.119** ***p (η***_**p**_^**2**^**) = 0.017 (0.10)**	*F* = 0.459 *p (η*_p_^2^) = 0.501 (0.01)	*F* = 1.966 *p (η*_p_^2^) = 0.167 (0.04)	***F* = 4.906** ***p (η***_**p**_^**2**^**) = 0.031 (0.09)**	*F* = 0.223 *p (η*_p_^2^) = 0.639 (0.00)	*F* = 0.750 *p (η*_p_^2^) = 0.390 (0.01)

0.02 ≤ *η*_p_^2^ ≤ 0.12 indicates small, 0.13 ≤ *η*_p_^2^ ≤ 0.25 indicates medium, and *η*_p_^2^ ≥ 0.26 indicates large effects. Bold values indicate statistically significant differences (*p* < 0.05)

### Emotional state outcomes

#### Balance confidence

There were significant main effects for group, threat, and arm, with participants (particularly in children) reporting lower balance confidence during the threat condition (irrespective of arm movement strategy) and during the restricted arm movement condition (irrespective of threat condition). The interaction effects between these factors were not statistically significant.

#### Fear of falling

Again, we detected significant main effects for group, threat, and arm but no significant interactions between these factors. The participants (particularly in children) reported greater fear of falling during the threat condition (irrespective of arm movement strategy) and during the restricted arm movement condition (irrespective of threat condition).

#### Perceived safety

There were significant main effects of group and threat but not of arm, with participants (particularly in children) stating lower perceived safety during the threat condition (irrespective of arm movement strategy). Further, we observed a significant group × arm interaction. *Post-hoc* tests revealed a significant decrease in perceived safety during walking with restricted compared to free arm movements (irrespective of threat condition) in children (no threat: *p* < 0.001, *d* = 0.24; threat: *p* = 0.008, *d* = 0.24) but not in young adults (no threat: *p* = 0.081, *d* = 0.29; threat: *p* = 0.210, *d* = 0.21). All other interactions did not reach the level of statistical significance.

#### Conscious balance processing

Again, we observed significant main effects for group and threat but not for arm, with participants (particularly in children) rating greater conscious balance processing during the threat condition (irrespective of arm movement strategy). No significant interaction effects were found.

### Walking outcomes

#### Gait speed

There were significant main effects threat and arm but not for group, with participants walking slower during the threat condition (irrespective of arm movement strategy) and during the restricted arm movement condition (irrespective of threat condition). The interaction effects between these factors were not statistically significant.

#### Cadence

There was a significant main effect of threat but not of group and arm, with participants showing lower cadence values during the threat condition (irrespective of arm movement strategy). Further, we observed a significant group × threat interaction. *Post-hoc* tests yielded a significant decrease in cadence when walking above ground than on ground level in young adults (free: *p* = 0.031, *d* = 0.45; restricted: *p* = 0.022, *d* = 0.38) but not in children (free: *p* = 0.123, *d* = 0.22; restricted: *p* = 0.052, *d* = 0.31). All other interactions did not reach the level of statistical significance.

## Discussion

This is the first study to compare the impact of free and restricted arm movements on subjective and objective indicators related to walking on ground (no threat) and at height (threat) in healthy children versus young adults. In line with our first hypothesis stating detrimental effects of height-induced postural threat and previous research (Brown et al. 2002; Gage et al. 2003; McKenzie and Brown 2004; Ellmers and Young 2018; Kluft et al. 2020), walking above ground level led to deteriorations in gait data and emotional state responses. Specifically, walking at a balance beam 80 cm above the ground resulted in decreased gait speed (in both age groups) and cadence (only in young adults). These findings imply the adoption of a more conservative gait behaviour and a more cautious strategy to compensate for the increased consequences of a fall related to walking at height. Alternatively, the observed changes in gait behaviour can be explained by the fact that not the height per se causes the destabilization but the accompanied emotional response to the postural threat. This explanation is supported by the observation that, in addition to the gait variables, the emotional responses were also negatively altered. Even before walking at height, participants rated their balance confidence lower and subsequently showed higher values for fear of falling and conscious balance processing and worse values for perceived safety. From a practical perspective, the gradual increase in walking height represents a promising opportunity to restore awareness of a previously automated gait behaviour and to focus attention to a cautious movement execution. In addition, this could lead to a more conscious balance processing combined with an increased perception of gait instabilities and fall-related anxiety.

Furthermore, restriction of arm movements negatively affected objective and subjective markers related to walking, as evidenced by a decrease in gait velocity, a decrease in balance confidence, and an increase in fear of falling. These observations are consistent with previous research (Hill et al. 2023; Lambrich et al. 2025) reporting deteriorations in postural control and emotional state outcomes during restricted arm movement conditions. For instance, Lambrich et al. (2025) asked young adults to walk with two different arm positions, i.e., free and restricted arm movements. Walking with restricted arm movements resulted in a significant decrease in gait speed and an increase in step time and was accompanied with a reduced balance confidence. These results indicate a cautious strategy aimed at increasing postural stability because the participants were not allowed to use their arms to counter any destabilisation. However, if arms can be moved freely, then a cautious strategy is not necessary because compensatory movements are possible. Precisely, free arm movements allow corrections of postural control aimed to (a) better maintain the centre of mass over the base of support (Roos et al. 2008), (b) increase the moment of inertia (Boström et al. 2018), (c) reduce angular acceleration (Hill et al. 2019), (d) generate counter-movements (Marigold et al. 2003), and (e) compensate for gravity-induced torques (Patel et al. 2014). For practitioners, the restriction of arm movements represents an easy-to-implement opportunity for exercise progression that leads to challenges in postural control, as would be the case in situations with an increased fall risk.

However, the detrimental effects of postural threat on subjective and objective outcomes related to walking were not amplified under restricted compared to free arm movement conditions. The observation of no threat by arm interaction is contrary to our second hypothesis and contradicts previous research (Hill et al. 2023; Lambrich et al. 2025) on that topic. For both, the static balance task (i.e., tandem stance on a raised surface) applied by Hill and colleagues and the dynamic balance task (i.e., 5 m walk on a raised beam) used by Lambrich and coworkers, the threat-related deteriorations in postural control and emotional state outcomes were more pronounced when arm movements were restricted. Methodological differences are most likely responsible for this discrepancy with the present study. Although Lambrich et al. (2025) used the same walking task, the gait variables were recorded using biomechanical methods (i.e., plantar pressure-detecting insoles). In the present study, on the other hand, only clinical methods (i.e., manually stopped walking time) were used, which have a lower measurement accuracy. Therefore, possible differences between free compared to restricted arm movements while walking at height may have remained undetected due to greater data dispersion.

Partly in agreement with our third hypothesis stating age differences, we detected differences between children and young adults in some subjective and objective outcomes related to walking. Specifically, children contrary to young adults showed no decrease in walking cadence from the no threat to the threat condition (irrespective of arm movement condition) and a decrease in perceived safety when walking with restricted compared to free arm movements (irrespective of threat condition). The decrease in walking cadence observed in young adults when walking at height compared to ground is consistent with the results of Lambrich et al. (2025) and indicates a more deliberate, controlled walking strategy, possibly reflecting greater caution or increased reliance on conscious balance control. The lack of such an adaptation in children was shown for the first time in this study and suggests a potentially less effective and maladaptive walking strategy. More specifically, the absence of modifications in walking cadence in children suggests that they may lack the ability to translate perceived threat into effective motor adjustments, leaving them potentially more vulnerable to imbalance or falls. Preliminary evidence for an opposite behaviour in children compared to young adults was already reported by Hill et al. (2024) for static postural control. Precisely, young adults showed a “stiffening” response (i.e., increased sway frequency and reduced sway amplitude) to a height-induced postural threat (bipedal stance on an elevated platform) but children demonstrated the opposite, i.e., reduced sway frequency and increased sway amplitude. Thus, the present findings extend existing research (Brown et al. 2002; Gage et al. 2003; McKenzie and Brown 2004; Hill et al. 2024) on age differences in postural threat to dynamic balance tasks and provides new insights into understanding how children adapt to walking in a hazardous situation. The potentially less effective and maladaptive walking strategy detected in children is most likely due to maturational deficits in the ability to control posture in children (Shumway-Cook and Woollacott 1985; Hirabayashi and Iwasaki 1995; Orendorz-Fraczkowska and Kubacka 2019; Sinno et al. 2021). For example, Orendorz-Frączkowska and Kubacka (2019) used the modified Clinical Test for the Sensory Interaction on Balance (mCTSIB) and tested children of different age groups (i.e., 6–7, 8–9, 10–11, 12–13, 14–15, and 16–17 years) under altered sensory conditions. Their findings indicate that when standing with eyes closed (i.e., proprioception dominant), younger children (6–11 years) exhibited faster sway velocities compared to older age groups. Similarly, when standing on a foam surface (i.e., visual input dominant), postural velocity was higher in 6- to 13-year-old children compared to those aged 14–17 years. Under conditions that primarily challenged vestibular function (i.e., eyes closed on foam surface), younger children (6–11 years) again displayed greater postural instability. Furthermore, Sino et al. (2021) applied the Sensory Organization Test (SOT) to compare sensory contributions to postural control between children (5–17 years) and young adults (20–25 years). They showed that somatosensory function developed early and was comparable to young adults around 5–8 years. Visual function follows, developing fully by approximately 12–14 years, while vestibular function developed latest, attaining adult-like characteristics around 15–17 years. Collectively, these findings indicate that there are marked developmental changes with respect to postural control in children compared to young adults, reinforcing the need to consider sensory system maturation when interpreting balance-related behaviours in younger populations. Alternatively, the lack of changes in walking cadence observed in children can be explained by the fact that height does not represent a postural threat per se but is only perceived as such when arm movements were restricted. This interpretation is supported by the finding that when walking at height, cadence increased during free arm movements but decreased during restricted arm movements.

The decrease in perceived safety shown by children, in contrast to young adults, when walking with restricted arm movements is also indicative of an incomplete maturation of postural control mechanisms. Consequently, challenging situations like walking with restricted arm movements are perceived differently than by young adults and led to a stronger emotional response (i.e., decreased perceived safety). Therefore, when assessing children's dynamic balance performance, practitioners should be aware that the restriction of arm movement can elicit a lower perceived safety, which can lead to misinterpretations of balance test outcomes.

The present study has some limitations that should be considered. Firstly, we did not apply objective physiological measurements of arousal/anxiety (e.g., electrodermal activity, heart rate) or additional anchors on the VAS scales, which limits the interpretation of the present results. Further studies are needed to refine our understanding about the effects of different arm movement strategies on emotional state and walking outcomes during height-induced postural threat. Secondly, no kinematic analyses of the arm movements were conducted. Thus, how and to what extent the upper body contributes to dynamic postural control remains unknown. Thirdly, our subjects were not harnessed, and we used a height of the balance beam (80 cm) that was rather low compared to the study of Ellmers and Young (2018) (i.e., 110 cm), which may have limited the magnitude of emotional and subsequent behavioural responses. We hypothesize that greater balance beam heights would lead to further decrements in emotional state and walking outcomes, which needs to be examined in future studies. Fourthly, we did not apply more robust and objective gait assessments, such as motion capture-based analyses of gait speed and cadence. The use of less precise assessments may have limited the accuracy of gait characterization. Future research should consider utilizing advanced technologies to achieve a more accurate and detailed assessment of gait parameters. Fifthly, to ensure equal conditions across both age groups, we did not provide additional explanations for the questionnaires to the children. As a result, we cannot fully exclude the possibility that the observed differences between children and young adults were influenced by varying levels of comprehension of the more abstract questionnaire items, rather than solely reflecting age-related differences in emotional responses. However, children's emotional responses to height-induced postural threat appear to be a relatively robust phenomenon. This is evidenced by the fact that the response pattern in the present study (where children did not receive additional explanation) closely aligns with the findings of Hill et al. (2024) where children were provided with additional clarification.

## Conclusions

The premise of our work was to investigate the effects of arm movements (free vs. restricted) on gait and emotional state outcomes during walking on ground (no threat) and above ground-level (threat) in children compared to young adults. Walking at height or with restricted arm movements independently led to deteriorations in gait (i.e., reduced speed) and emotional state (i.e., decreased balance confidence and increased fear of falling) outcomes. The additionally observed age differences (e.g., children did not reduce their cadence when walking at height) suggest a potentially less effective and maladaptive walking strategy in children (presumably due to immaturities in postural control mechanisms).

## Data Availability

All data generated or analysed during this study are included in this published article.
